# Understanding the Trajectory to a Diagnosis of Tetanus: A Descriptive Study

**DOI:** 10.7759/cureus.33287

**Published:** 2023-01-03

**Authors:** Yuji Okazaki, Toshihisa Ichiba, Noritomo Fujisaki, Seigo Urushidani

**Affiliations:** 1 Emergency Medicine, Hiroshima City Hiroshima Citizens Hospital, Hiroshima, JPN; 2 Emergency and Critical Care Center, Kurashiki Central Hospital, Kurashiki, JPN

**Keywords:** visited department, trismus, neck stiffness, dysphagia, tetanus

## Abstract

Background and objective

Tetanus is a rare but potentially fatal disease in developed countries, including Japan. It is very difficult to diagnose this condition early because of its broad symptomatology and the lack of familiarity with tetanus among both physicians and patients. In this study, we aimed to describe the clinical manifestations of tetanus and to examine as to which departments/branches of medicine patients consult in order to provide helpful information for diagnosing this challenging condition.

Materials and methods

This was a descriptive, retrospective study conducted at a single tertiary hospital from January 2011 to December 2021. Patients with generalized tetanus, cephalic tetanus, and local tetanus presenting to our emergency department were included in the study. We examined the clinical manifestations and departments that patients with tetanus visited first. Additionally, the initial diagnosis and diagnostic evaluation before the diagnosis were examined.

Results

Of the total 10 patients included in the study, nine had generalized tetanus and one had cephalic tetanus; the common initial manifestations were neck stiffness (30%), dysarthria (20%), and trismus (20%). Of note, 80% of patients also complained of dysphagia before the diagnosis. Patients first visited various departments, including a dental clinic (30%) and the department of otorhinolaryngology (20%). Only two patients were accurately diagnosed with tetanus at the first department they visited. Physicians performed head imaging for all the patients.

Conclusions

Based on our findings, in Japan, patients with tetanus present with symptoms that physicians interpret or suspect to be associated with disorders of the central nervous system. Meanwhile, patients themselves tend to consider the symptoms as indicative of oral or dental issues. Further prospective studies involving a larger number of participants are needed to investigate the clinical course of tetanus until the establishment of a diagnosis.

## Introduction

Tetanus is caused by a neurotoxin produced by the common soil bacterium Clostridium tetani. Although this disease is rare in developed countries because of active immunization with tetanus toxoid [1,2], it is still life-threatening [3,4]. In Japan, approximately 50-100 cases are reported every year, predominantly in the elderly population because of the decreased effects of vaccination due to the failure to receive the booster vaccine or the lack of basic immunity due to an inadequate vaccination system in childhood [4]. Due to the global population aging and the increase in natural disasters due to climate change, it is important to better understand tetanus, even in developed countries that have a low prevalence of tetanus [5].

The clinical diagnosis of tetanus involves accurately identifying its typical symptoms including trismus, muscle rigidity, and painful spasm [6]. However, since most physicians in developed countries have no experience with diagnosing tetanus, they are sometimes not aware of it even when patients present with classic symptoms of the disease [1,7]. In addition, in developed countries with free access to medical facilities and few immigrants from developing countries, such as Japan, patients with tetanus may not know which specialty to first approach when seeking care as the patients themselves are unaware of this rare disease. Thus, it can be very challenging for physicians to diagnose this condition, especially in the period soon after its onset [8,9].

Symptoms of tetanus can vary over a period of hours to days, but how they appear and change is not well known. In addition, in developed countries, information is scant as to which departments patients should visit for obtaining a proper diagnosis of tetanus. The purpose of this study was to describe the clinical manifestations of patients diagnosed with tetanus and to examine what specialties/departments patients visit for a tetanus diagnosis based on the changes in their symptoms. A detailed investigation of patient trajectory to a diagnosis will provide important clues to diagnosing tetanus more promptly and in a timely manner in countries with a low prevalence of tetanus.

## Materials and methods

Study design and setting

This was a descriptive, retrospective study conducted at a tertiary hospital between January 2011 and December 2021. Our hospital is located in the center of Hiroshima city, and our emergency department receives approximately 7,000 ambulances and more than 15,000 walk-in patients annually. The requirement of informed consent was waived because the data used were anonymous and we employed an opt-out recruitment approach. This study was approved by the Institutional Review Broad of Hiroshima City Hiroshima Citizens Hospital (approval no: 2022-15) and was conducted in accordance with the Strengthening the Reporting of Observational Studies in Epidemiology (STROBE) guidelines [10].

Study participants

We included all consecutive patients aged ≥18 years who visited our emergency department and were classified to have a diagnosis of tetanus in the electronic medical records. The diagnosis of tetanus was based on clinical manifestations and physical examinations because culture on injury sites was not conducted for all patients due to uncertainty about the sites of injury [6]. The clinical manifestation included generalized or localized muscle spasms and painful spasms, and physical examinations revealed muscle rigidity. We included three types of tetanus in this study: generalized tetanus, cephalic tetanus, and local tetanus. One author (YO) checked the electronic medical charts and excluded patients who were not considered as cases of tetanus based on the comprehensive approach toward diagnosing tetanus. We also excluded other causes of generalized muscle spasms including strychnine poisoning and dystonic reactions to drugs.

Measures

Information on patient age, sex, types of tetanus, comorbidities, traumatic events, vaccination, and disease severity were collected from the electronic medical records. The severity of tetanus was defined based on the Ablett classification [11]. The severity was classified into four grades, ranging from 1 to 4. While grades 1 and 2 represented mild or moderate conditions that did not require mechanical ventilation, grades 3 and 4 represented severe conditions that required mechanical ventilation. Grade 4 was the most severe condition and was associated with autonomic dysfunction.

Outcomes

At the outset, we examined the initial manifestations and determined which departments patients with tetanus visited first. Clinical manifestations were also focused on. Regarding the visited departments, we counted the departments that patients visited before the diagnosis irrespective of whether they are at our hospital or not. Secondly, we examined the initial diagnosis at the department that the patient visited first and diagnostic examinations before the diagnosis was made. Third, we examined the differences in time to diagnosis and clinical outcomes between patients with and without a delayed diagnosis. A delayed diagnosis was defined as cases where patients were not accurately diagnosed with or not suspected to have tetanus at the department they visited first. Regarding time to diagnosis, we measured this parameter in units of days based on two periods: the duration from the onset of tetanus to the visit to the first department, and the duration from the onset of tetanus to receiving a diagnosis. Regarding clinical outcomes, we evaluated the following variables: intubation, tracheostomy, discharge to other facilities, length of stay (LOS) in the hospital, and mortality in the hospital. These outcomes, except for LOS in the hospital, were measured in terms of the proportion of patients, while LOS in the hospital was measured in units of days.

Statistical analysis

Data on categorical variables were presented as proportions, and data on continuous variables for non-normal distribution based on a histogram were summarized in median and interquartile ranges (IQR). Regarding outcomes of interest, we calculated the proportion with the number of outcomes of interest in patients with tetanus divided by the total number of patients. We compared categorical variables using Fisher’s exact test, and continuous variables using a Wilcoxon rank-sum test between patients with and without a delayed diagnosis. There were no missing data on the characteristics of patients and outcomes of interests. All analyses were performed using STATA/MP-Parallel Edition version 16.1 (StataCorp LLC, College Station, TX). Two-sided p-values <0.05 were considered statistically significant.

## Results

A total of 10 patients were included based on the inclusion and exclusion criteria. Table 1 summarizes the patient characteristics. The median age of patients with tetanus was 77.5 years (IQR: 70-81), and six (60%) of them were women. Nine (90%) patients were diagnosed with generalized tetanus, and none of the patients were diagnosed with local tetanus. Five (50%) had grade 3 disease or higher per the Ablett classification.

**Table 1:** Characteristics of patients with tetanus IQR: interquartile range

Variables	Values
Age in years, median (IQR)	77.5 (70-81)
Females, n (%)	6 (60)
Types of tetanus, n (%)	
Generalized tetanus	9 (90)
Cephalic tetanus	1 (10)
Local tetanus	0
Comorbidities, n (%)	
Hypertension	4 (10)
Hyperlipidemia	3 (30)
Rheumatic arthritis	1 (10)
Basedow disease	1 (10)
Cerebral infarction	1 (10)
Coronary artery disease	1 (10)
Traumatic events, n (%)	5 (50)
Vaccination within 10 years, n (%)	
None	3 (30)
Unclear	7 (70)
Ablett classification, n (%)	
1	0
2	5 (50)
3	2 (20)
4	3 (30)

**Figure 1:**
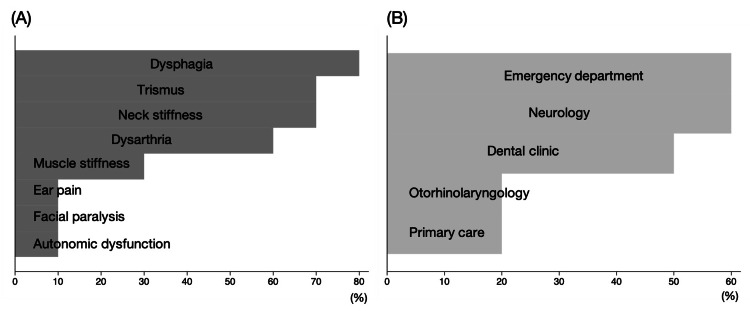
Clinical manifestations (A) and departments visited (B) before the diagnosis of tetanus The figure shows the proportion of each clinical manifestation (A) and departments visited (B) before a diagnosis of tetanus in the total study participants

Table 2 shows the first manifestations and departments that patients with tetanus visited first. The most common first manifestation was neck stiffness (3/10; 30%), followed by dysarthria (2/10; 20%) and trismus (2/10; 20%). In addition, while four (40%) patients visited an emergency department first, two (20%) of the patients visited the department of otorhinolaryngology first, three (30%) visited the dental clinic, and one (10%) first visited the primary care clinic (Table 2). Figure 1 shows the clinical manifestations and departments visited before the diagnosis. Most of the patients complained of dysphagia (8/10; 80%), trismus (7/10; 70%), and neck myalgia (7/10; 70%) before the diagnosis (Figure 1A). We also describe the chief complaints as stated in the medical questionnaire upon arrival to our emergency department in the Appendices (Table 5). As shown in Figure 1B, five (50%) patients visited the dental clinic before the diagnosis.

**Table 2:** First manifestations of tetanus and the departments that patients visited first

First manifestation	Number of patients (%)	Department visited first	Number of patients (%)
Neck stiffness	3 (30)	Emergency department	4 (40)
Trismus	2 (20)	Dental clinic	3 (30)
Dysarthria	2 (20)	Otorhinolaryngology	2 (20)
Dysphagia	1 (10)	Primary care clinic	1 (10)
Ear pain	1 (10)		
Facial paralysis	1 (10)		

At the department visited first, only two (20%) patients were diagnosed with tetanus (Table 3). In this study, tetanus was misdiagnosed as a primary headache, temporomandibular joint disorder, and Bell’s palsy. Physicians performed a CT of the head (8/10; 80%) and MRI of the head (5/10; 50%) before the diagnosis (Table 3), which suggests that disorders of the central nervous system including acute stroke were suspected.

**Table 3:** Initial diagnosis made at the department visited first and diagnostic examination performed before the diagnosis CT: computed tomography; MRI: magnetic resonance imaging

Diagnosis	Number of patients (%)	Examination before diagnosis	Number of patients (%)
Tetanus	2 (20)	Head CT	8 (80)
Primary headache	1 (10)	Facial or neck CT	4 (40)
Temporomandibular joint disorder	2 (20)	Head MRI	5 (50)
Bell’s palsy	1 (10)	Lumbar puncture	1 (10)
Uncertain	4 (40)		

Table 4 shows the time to diagnosis and clinical outcomes between patients with and without a delayed diagnosis. The median time from the onset of symptoms to visiting the first department was not significantly different between patients with and without a delayed diagnosis [3.25 days (IQR: 1.5-5) and three days (IQR: 1-4.5), respectively; p=0.591]. Also, the median time from the onset to the diagnosis was similar between these two subsets of patients [5.63 days (IQR: 4.38-7.25) and 3.25 days (IQR: 1.5-5), respectively; p=0.295]. In addition, the occurrence of clinical outcomes, which were intubation, tracheostomy, discharge to other facilities, and mortality in the hospital, was not significantly different between patients with and without delayed diagnosis (Table 4). There was also no difference in LOS in the hospital between patients with and without delayed diagnosis [median: 52 days (IQR: 10-94) and 31.5 days (IQR: 26-41.5), respectively; p=0.896].

**Table 4:** Comparison of time to diagnosis and clinical outcomes between patients with and without a delayed diagnosis *Wilcoxon rank-sum test was conducted to evaluate the difference between patients with and without a delayed diagnosis ^†^Fisher’s exact test was conducted to examine the difference between patients with and without a delayed diagnosis IQR: interquartile range

Variables	Total patients (n=10)	Patients without delayed diagnosis (n=2)	Patients with delayed diagnosis (n=8)	P-value
Time from symptom onset to visiting the first department, days, median (IQR)	3 (1-5)	3 (1-4.5)	3.25 (1.5-5)	0.591^*^
Time from symptom onset to diagnosis, days, median (IQR)	4.89 (4.25-6.5)	3.25 (1.5-5)	5.63 (4.38-7.25)	0.295^*^
Intubation, n (%)	5 (50)	1 (50)	4 (50)	0.778^†^
Tracheostomy, n (%)	4 (40)	1 (50)	3 (37.5)	0.667^†^
Discharge to other facilities, n (%)	4 (40)	1 (50)	3 (37.5)	0.667^†^
Length of stay in the hospital, days, median (IQR)	31.5 (26-48)	52 (10-94)	31.5 (26-41.5)	0.896^*^
Mortality in the hospital	0	0	0	-

## Discussion

We found that six of the 10 patients first presented with neck stiffness, dysarthria, or dysphagia. Based on these symptoms, physicians suspected disorders of the central nervous system and performed head imaging. In addition, many patients visited the department of otorhinolaryngology or dental clinic before a diagnosis of tetanus was made. It took approximately five days to arrive at a diagnosis of tetanus and the number of patients with a delayed diagnosis was substantial, which suggests that it was difficult to suspect and diagnose tetanus until typical symptoms appeared. There was no association between clinical outcomes and delayed diagnosis of tetanus in this study.

To the best of our knowledge, this is the first study to focus on the first manifestations and departments visited first among patients with tetanus in countries with a low prevalence. Some studies have reported that the most common symptom was trismus, but these studies examined the symptoms on hospital admission [12-14]. In the literature in Japanese on the clinical characteristics of tetanus, 6/11 (55%) patients did not present with trismus as an initial symptom, but the investigation did not focus on the visited department before the diagnosis [15]. Our findings show both the clinical manifestations and the visited departments among patients with tetanus. This can be helpful for developing a better understanding of the clinical course of tetanus before reaching a diagnosis in countries with a low prevalence.

Patients with tetanus may present with neck stiffness, dysphagia, or dysarthria immediately after the disease's onset, while trismus is also known as a familiar symptom. These symptoms of tetanus are caused by the tetanus toxin (i.e., tetanospasmin), which is produced by Clostridium tetani [16]. After reaching the brainstem and spinal cord, the toxin results in the inactivation of inhibitory neurotransmission that normally modulates anterior horn cells, causing muscle contractions. As a result, affected patients experience increased muscle tone and painful spasms [16,17]. Due to this damage mechanism by the tetanus toxin, dysphagia and dysarthria may occur because of the effects on the glossopharyngeal nerve and the vagus nerve, with neck stiffness resulting from the effects on accessory nerves, and trismus occurring due to effects on the trigeminal nerve and the facial nerve. We hypothesized that it is more important to determine what symptoms patients complain of as subjective symptoms than to find which cranial nerves are more susceptible in patients with tetanus. In other words, while the cranial nerves may be equally affected by the tetanus toxin, patients are less likely to complain of trismus as an initial symptom as in our study although this may be present as a physical finding from the early onset of the disease [18]. Thus, physicians should consider whether patients with neck stiffness, dysphagia, or dysarthria present with impaired mouth opening.

It may be more difficult to accurately diagnose tetanus at the department that patients visit first in countries with a low prevalence of tetanus compared to countries where tetanus is frequently diagnosed. There are two reasons for this: (1) in countries with free access to medical facilities, patients choose by themselves which departments to first visit according to their symptoms, and (2) physicians may suspect acute stroke or meningitis based on their symptoms because tetanus is extremely rare and physicians have probably never diagnosed this condition. According to the results of our study, neither the patients nor physicians may be familiar with tetanus. Interestingly, half of the patients visited a dental clinic before a diagnosis. On the other hand, in countries where tetanus is frequently diagnosed, physicians may suspect tetanus even in the presence of mild symptoms (e.g., impaired mouth opening or neck stiffness), and patients themselves may recognize that their symptoms are due to tetanus if the disease progresses. Thus, it is important to understand what departments patients visit according to their symptoms in order to diagnose this condition as early as possible, especially in countries where tetanus is rarely diagnosed, such as Japan.

Delayed diagnosis might not be associated with clinical outcomes in situations where physicians and patients are unfamiliar with tetanus. This study shows that the duration from symptom onset to visiting the first department was two days shorter than the duration from symptom onset to diagnosis among patients with a delayed diagnosis. This suggests that the time to diagnosis may depend on the time till typical symptoms appear. Also, the time till typical symptoms appear may be affected by the immunity to tetanus. However, the seroprevalence of tetanus toxoid antibody (>0.1 IU/ml) in Japan is less than 5% among people aged 65 years and older [19]. In this study, all patients with tetanus were elderly individuals, which may limit the impact on the results. Based on previous literature, early and prompt treatment such as antibiotics and human tetanus immune globulin may improve clinical outcomes [20]. The difference between the results of this study and those of previous literature pertains to variations in the immunity of patients and the amount of tetanus toxin. Thus, further studies are needed to determine whether earlier treatment for tetanus, before typical symptoms appear, can prevent death and complications in countries with a low prevalence of tetanus.

Several limitations of this study should be acknowledged. First, the number of study participants was small, and this study was conducted at a single center. The scope for external validation of our findings may be limited. Secondly, an adequate sample size could not be calculated because there were no previous studies that investigated the relationship between a delayed diagnosis and clinical outcomes for us to rely on. Also, when comparing diagnoses with and without a delay, we could not adjust the clinical outcomes for confounding factors because of the small sample size. Third, we could not measure the amount of toxin and degree of immunity in included patients in this study. These factors may affect the severity of tetanus and may also be associated with the initial symptoms. Future studies should incorporate these important variables. Finally, an element of information bias may have crept in when the symptoms of patients were extracted from the medical record. We extracted the symptoms that physicians or nurses obtained through interviews with patients, but did not categorize the symptoms based on a predefined protocol. Thus, a prospective study using predefined categories of symptoms would be required to resolve this issue of bias.

## Conclusions

Based on our findings, patients with tetanus in Japan first present with symptoms that usually prompt physicians to suspect disorders of the central nervous system in these patients, while patients themselves tend to consider these symptoms as indicative of oral or dental issues. Physicians need to be aware of the clinical manifestations of tetanus and the departments that patients visit before a diagnosis in order to accurately diagnose tetanus. Further prospective studies are needed to examine in detail the time course from the onset of symptoms to diagnosis among a larger number of study participants.

## Appendices

**Table 5:** Chief complaints as stated in the medical questionnaire at the emergency department by included patients with tetanus GT: generalized tetanus; CT: cephalic tetanus

Age, years	Sex	Diagnosis	Chief complaints
93	Woman	GT	I can no longer speak as well as I would like
70	Woman	GT	I cannot open my mouth
69	Man	GT	I cannot eat anything
77	Woman	GT	I cannot move my neck because it hurts
78	Man	GT	I cannot turn my neck
75	Woman	GT	I have phlegm in my throat, and cannot get it out
82	Woman	GT	I cannot speak well
80	Woman	GT	I feel discomfort in my throat and cannot speak well
81	Man	GT	I cannot swallow a meal
65	Man	CT	I cannot open my mouth
